# Timescale- and Sensory Modality-Dependency of the Central Tendency of Time Perception

**DOI:** 10.1371/journal.pone.0158921

**Published:** 2016-07-12

**Authors:** Yuki Murai, Yuko Yotsumoto

**Affiliations:** 1 Department of Life Sciences, The University of Tokyo, Tokyo, Japan; 2 Japan Society for the Promotion of Sciences, Tokyo, Japan; Duke University, UNITED STATES

## Abstract

When individuals are asked to reproduce intervals of stimuli that are intermixedly presented at various times, longer intervals are often underestimated and shorter intervals overestimated. This phenomenon may be attributed to the central tendency of time perception, and suggests that our brain optimally encodes a stimulus interval based on current stimulus input and prior knowledge of the distribution of stimulus intervals. Two distinct systems are thought to be recruited in the perception of sub- and supra-second intervals. Sub-second timing is subject to local sensory processing, whereas supra-second timing depends on more centralized mechanisms. To clarify the factors that influence time perception, the present study investigated how both sensory modality and timescale affect the central tendency. In Experiment 1, participants were asked to reproduce sub- or supra-second intervals, defined by visual or auditory stimuli. In the sub-second range, the magnitude of the central tendency was significantly larger for visual intervals compared to auditory intervals, while visual and auditory intervals exhibited a correlated and comparable central tendency in the supra-second range. In Experiment 2, the ability to discriminate sub-second intervals in the reproduction task was controlled across modalities by using an interval discrimination task. Even when the ability to discriminate intervals was controlled, visual intervals exhibited a larger central tendency than auditory intervals in the sub-second range. In addition, the magnitude of the central tendency for visual and auditory sub-second intervals was significantly correlated. These results suggest that a common modality-independent mechanism is responsible for the supra-second central tendency, and that both the modality-dependent and modality-independent components of the timing system contribute to the central tendency in the sub-second range.

## Introduction

Interval timing in the millisecond-to-second range is vital for many human behaviors [1]. However, timing behavior is often susceptible to perceptual noise and decision uncertainty. Indeed, many psychophysical studies have indicated that timing systems use various clues to optimally estimate stimulus intervals [2–4]. In particular, a considerable number of researchers have focused on examining how interval timing relies on temporal and non-temporal contextual information, such as previously presented intervals and sensory modality, respectively (for reviews, [5, 6]). Vierordt's Law is a representative example of how contextual information can influence temporal processing. According to Vierordt's Law, when stimuli with various durations are intermixedly presented, longer durations are underestimated and shorter durations are overestimated [7]. One predominant explanation for this phenomenon is Hollingsworth's (1910) central tendency of judgment, in which the perception of time elements in a series is biased toward the mean of that series [8].

Several psychophysical studies have revealed that the magnitude of the central tendency differs substantially between individuals, and investigated which components of the timing system mediate these individual differences [9–11]. Studies that use computational modeling have shown that the central tendency is associated with timing precision; the noisier the internal representation of the interval, the larger the central tendency [12, 13]. As described below, timing precision may depend on various factors, including sensory modality and the length of the timed stimulus.

A traditional but widely accepted model for interval timing in the millisecond-to-second range is the pacemaker-accumulator model. This model assumes the presence of a modality- and timescale-invariant central clock [14]. The scalar property also assumes that timing precision, defined as the ratio of the standard deviation of the perceived interval to the stimulus interval, is constant in the millisecond-to-second range, and is a prominent finding in the field of time perception [15, 16]. Furthermore, recent modeling studies also suggest the presence of a modality- and timescale-independent timing system [17, 18]. In contrast, many psychological studies have indicated that timing precision does depend on the sensory modality [9] and timescale [19, 20]. Durations in the sub-second (i.e., hundreds of milliseconds) and supra-second (i.e., several seconds) ranges are involved in different behavioral functions, and therefore recruit different neural mechanisms [1]. For example, neuroimaging studies have demonstrated that there are two timing systems divided by a boundary of approximately one second [21, 22]. Moreover, several computational models of time perception include sensory modality dependent components, especially for sub-second timing, such as time-dependent changes in the state of the neural network [23, 24], or time-sensitive mechanisms in early sensory processing [3, 4]. In line with these studies, Cicchini et al. (2012) found that visually defined sub-second intervals induced a larger central tendency than auditorily defined sub-second intervals due to the higher temporal precision of the auditory modality. Whether such modality-dependent variations in the central tendency occur for supra-second intervals is still a matter of debate [25, 26].

The present study aimed to examine how stimulus modality affects the optimal encoding of time across different timescales. If the source of timing noise is located at modality-dependent timing mechanisms, then the central tendency for visual and auditory intervals should occur independently. If a common modality-independent timing mechanism is also involved in the central tendency, then the central tendencies for the visual and auditory modalities should show within-individual correlations. We hypothesized that the central tendency occurs differently for auditory and visual timing in the sub-second range and depends on modality-specific processing, whereas a common modality-independent timing system regulates the central tendency in the supra-second range. To test these hypotheses, we quantified the magnitude of the central tendency for visual and auditory timing in the sub- and supra-second ranges.

In Experiment 1, we investigated how stimulus modality and timescale affect individual differences in the central tendency. In Experiment 2, we examined whether modality-dependent time encoding in the sub-second range results from differences in temporal sensitivity between the visual and auditory systems by controlling for differences in the discrimination of intervals in the sub-second range between the two modalities.

## Experiment 1

### Materials and Methods

#### Participants

Twenty healthy volunteers (13 males and 7 females, 18–29 years old) participated in Experiment 1. All participants gave written informed consent for their participation in the experimental protocol, which was approved by the institutional review board at The University of Tokyo. All participants reported to have normal hearing and normal or corrected-to-normal vision.

#### Apparatus

The auditory stimuli were presented through an Audio Stream Input/Output (ASIO) compliant USB digital-to-analog converter (Roland UA-1G) and SONY MDR-XB500 headphones at 60 dB. The visual stimuli were presented on a CRT monitor (Mitsubishi Electric RDF223H, 1024 × 768 pixels, 120 Hz refresh rate). Participants were seated 57.3 cm from the monitor in a dark soundproof room; participants’ heads were stabilized using a chin rest.

#### Stimuli and procedure

**Fig 1:**
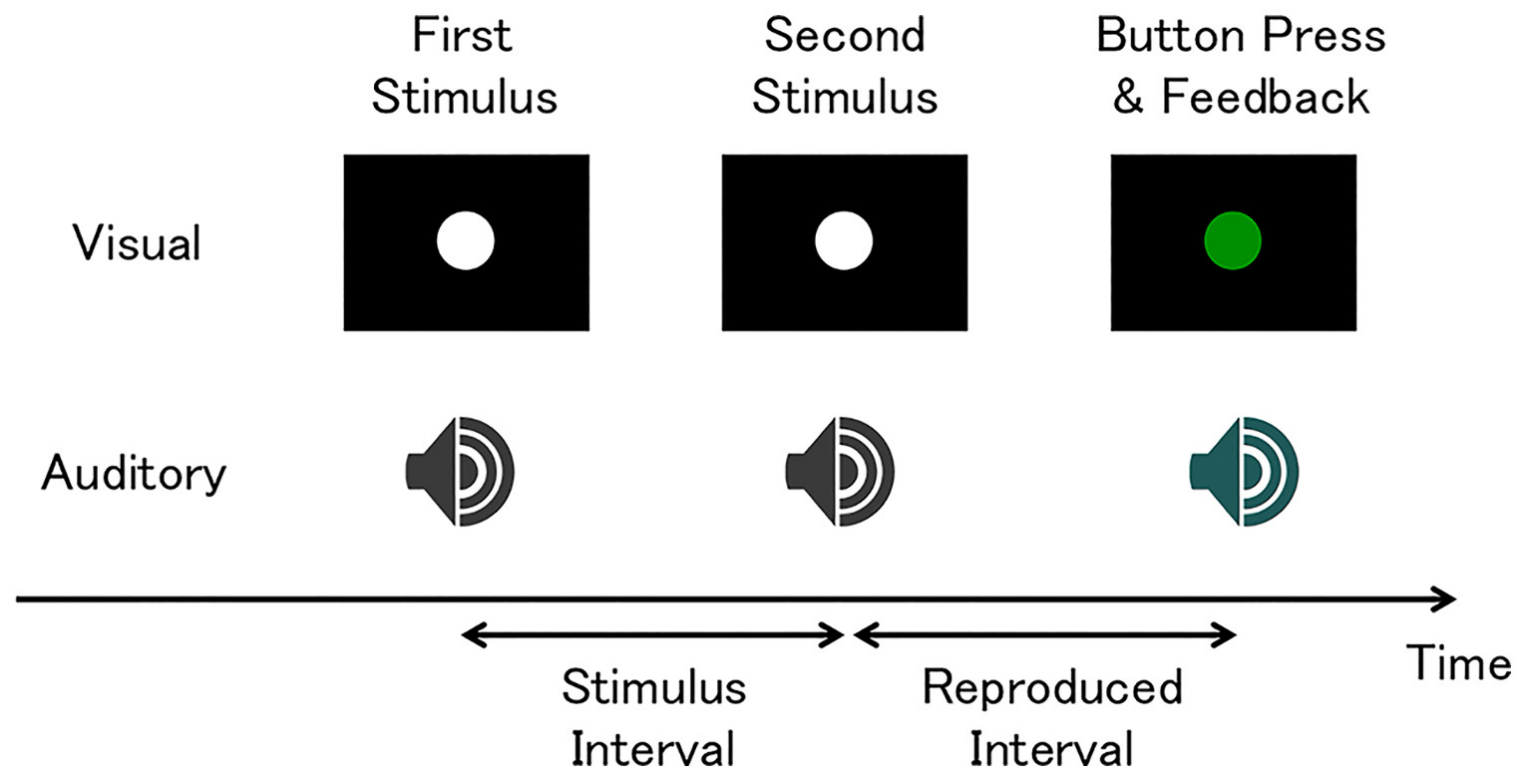
Schematic of the experimental procedure. Two brief flashes or tones were sequentially presented, and participants made button presses to reproduce the interval. Correct or incorrect feedback was presented immediately after participants’ responses.

Stimuli were generated using MATLAB (MathWorks, R2012b) and the Psychophysics Toolbox [27, 28]. A schematic of the task is shown in Fig 1. In the task, a pair of stimuli was briefly presented, one after the other. The visual stimulus was a white disk (5 degrees in diameter), while the auditory stimulus was a simple tone (600Hz, 60dB). Participants were asked to reproduce intervals between the pair of stimuli by pressing a button with the forefinger of their dominant hand. Participants were instructed not to count the duration between stimuli [29]. Each visual flash or auditory tone lasted for 20 ms. A cosine ramp of 5 ms was applied to the onset and offset of all auditory stimuli. Stimulus intervals were either sub-second or supra-second. The sub-second intervals were 400–600 ms with 50 ms steps. The supra-second intervals were 2000–3000 ms with 250 ms steps; the supra-second intervals were scaled 5 times longer. The inter-trial intervals (ITI) ranged from 1.2 s to 1.8 s.

Immediately after the subject’s response, a sensory feedback was given in every trial. If the reproduced interval was within a certain time frame of the actual interval, the same stimulus (i.e. a white disc or 600 Hz tone) was presented as positive (correct) feedback; otherwise, a green disk or a 400 Hz tone was presented as negative (incorrect) feedback. Each sensory feedback lasted for 20 ms. To compensate for the scalar property, the ratio of the width of this feedback time window to the stimulus interval was kept constant across the different stimulus intervals. This feedback ratio was adaptively controlled using a one-up, one-down staircase method that adds or subtracts 0.015 for each incorrect or correct trial, respectively [12].

Participants completed 4 separate experimental blocks, each of which was one of the 4 combinations of sensory modalities and timescales (i.e., visual sub-second, auditory sub-second, visual supra-second, auditory supra-second). At the beginning of each block, participants completed a practice session with 50 trials in order to become accustomed to the timescales and modalities. After the practice session, participants completed a total of 200 trials in each block. Each block was divided into 2 sessions for sub-second conditions, or 4 sessions for supra-second conditions. The presentation order of intervals within a session was randomized. The order of blocks was also randomized across participants using a Latin square method. Participants completed all 4 blocks twice by coming in on two separate experimental days. In total, 400 trials were completed for each timescale-modality condition.

#### Analysis

Trials in which the reproduced interval deviated more than 3 standard deviations (SDs) from each condition’s mean were excluded from all analyses. We linearly regressed the reproduced interval to the stimulus interval for each individual. The slopes of the linear regressions were compared across conditions as indices of the central tendency. For example, if stimulus intervals were reproduced accurately then the slope was 1. However, if the central tendency occurred and longer intervals were underestimated and shorter intervals overestimated, the slope values were less than 1. To examine how stimulus modality and timescale affect the central tendency, we conducted a two-way repeated-measures ANOVA with stimulus modality (visual or auditory) and timescales (sub- or supra-second) as factors. Furthermore, we calculated within-individual correlations of the central tendency across different sensory modalities and timescales, in order to investigate whether modality- and timescale-independent timing systems are involved in the central tendency.

### Results and Discussion

**Fig 2:**
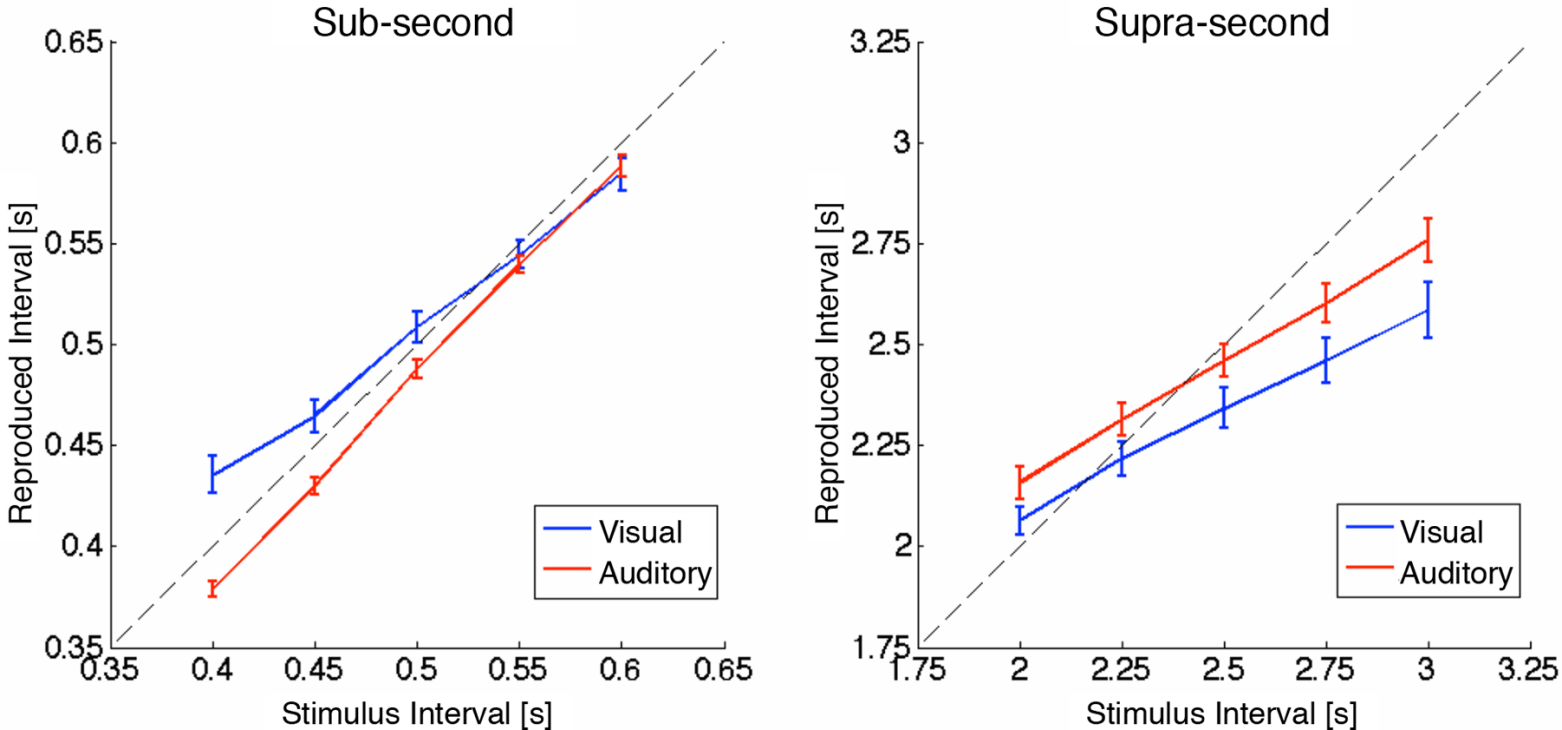
Group means of the reproduced intervals. The left panel indicates the results for sub-second timing. The right panel indicates the results for supra-second timing. Blue lines represent results for visual stimuli, and red lines represent results for auditory stimuli. Dotted lines correspond to accurate reproduction of the stimulus intervals. Error bars represent the standard error of the mean.

Fig 2 shows the reproduced intervals in each condition. Overall, the central tendency was observed at a group level in all of the conditions except the auditory sub-second condition, as shown in previous studies [9, 25].

**Fig 3:**
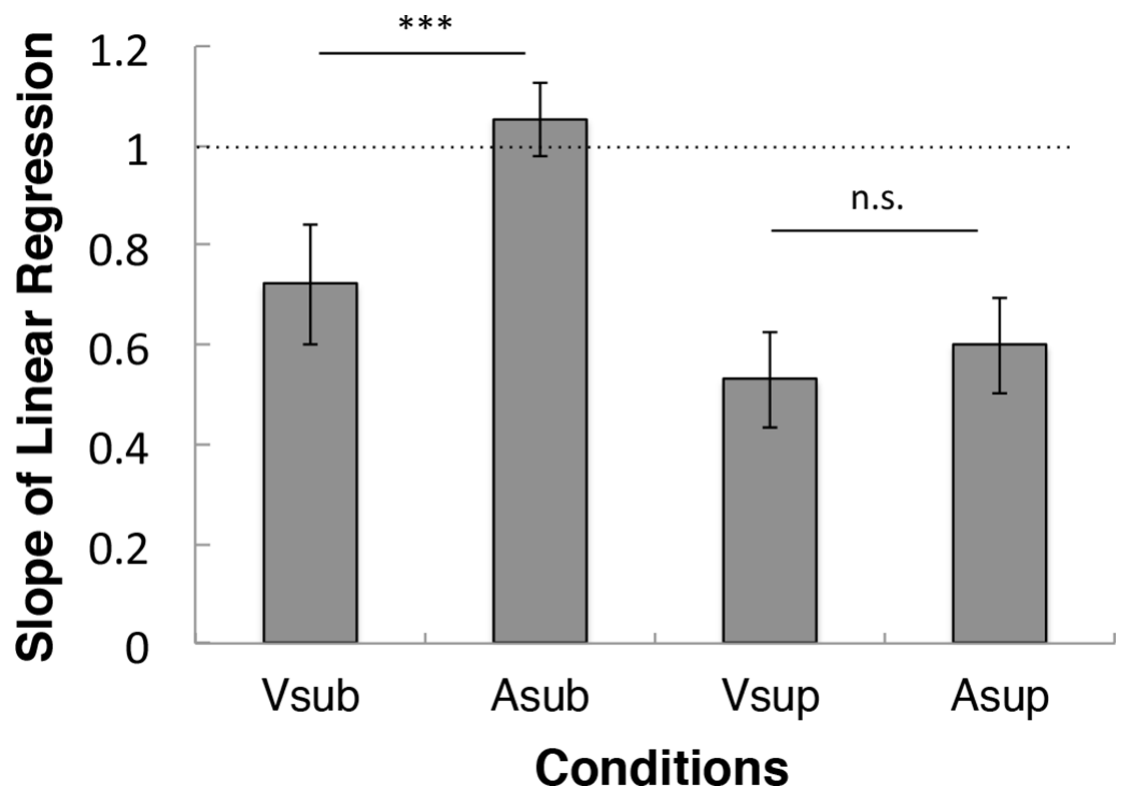
Quantification of the central tendency. The slopes of the linear regressions are presented for all four conditions. A and V represent auditory and visual stimuli, respectively. Sub and sup represent sub-second and supra-second intervals, respectively. Error bars represent the standard error of the mean. *** p < .001, n.s. = not significant.

Fig 3 shows the magnitude of the central tendency in each condition. A two-way repeated-measures ANOVA revealed significant main effects of the stimulus modality (F(1,76) = 17.5, p < .001) and timescale (F(1,76) = 55.8, p < .001), as well as a significant interaction (F(1,76) = 9.20, p = .003). These results indicate that (1) the visual modality is more susceptible to the central tendency, (2) the magnitude of the central tendency is larger in the supra-second range, and (3) the difference in the magnitude of the central tendency between visual and auditory intervals is larger in the sub-second range. As shown in Fig 3, the magnitude of the central tendency was significantly different between the visual and auditory modalities for sub-second timing (t(19) = 6.14, p < .001), but not supra-second timing (t(19) = 2.16, p = .087, Bonferroni-corrected; the Bonferroni corrections were applied for two comparisons).

**Fig 4:**
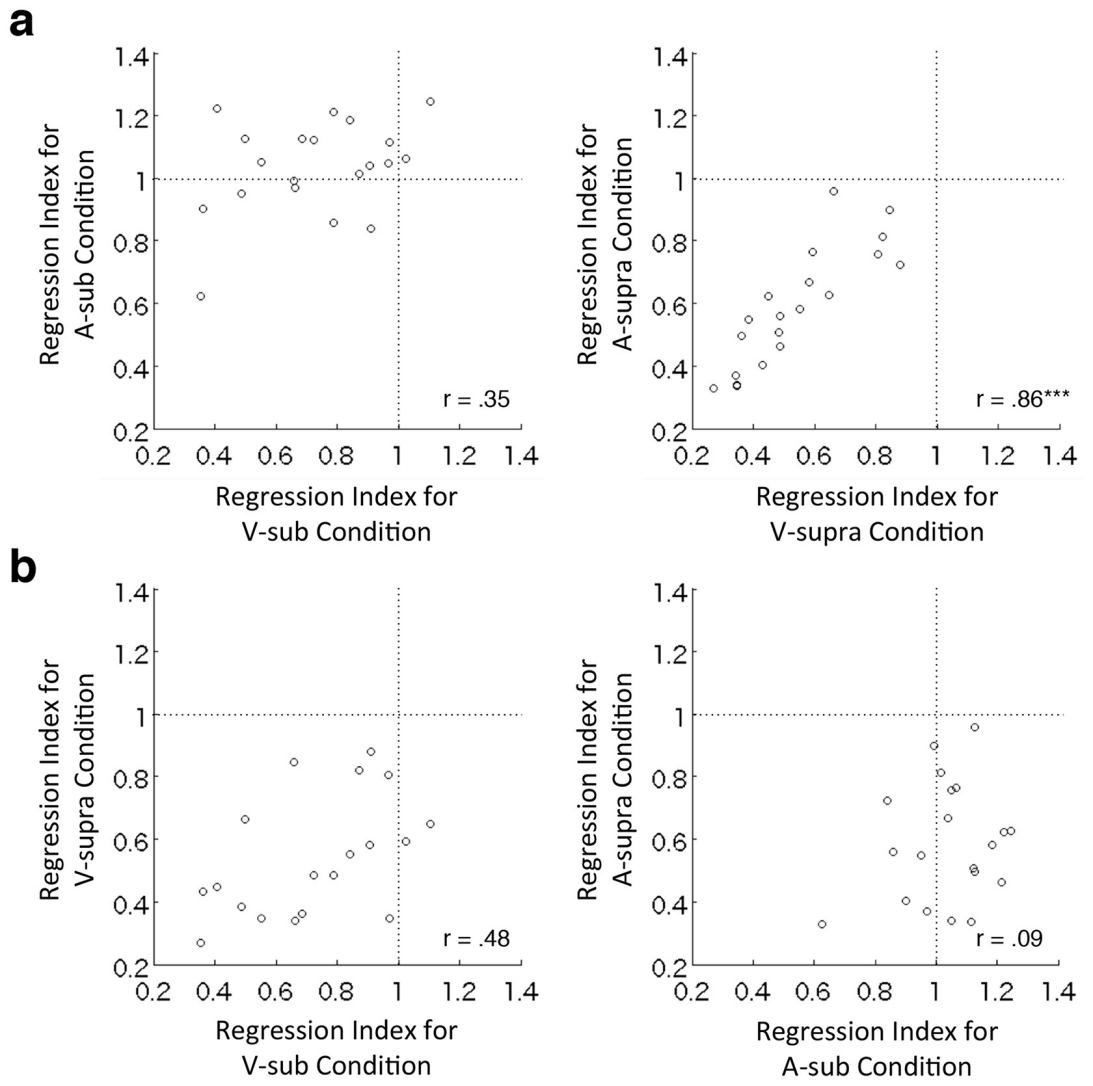
Within-individual correlations of the central tendency across different sensory modalities (a) and timescales (b). Regression index represents the slope of the linear regression of the reproduced intervals to the stimulus intervals. Each small circle represents an individual’s data. V and A stand for the visual and auditory conditions, respectively. Sub and supra represent the sub-second and supra-second intervals, respectively. *** p < .001.

We further examined within-individual correlations of the central tendency across modalities and timescales. The magnitudes of the central tendency for the visual and auditory modalities were significantly correlated for supra-second timing (r = .86, p < .001, Bonferroni-corrected), but not sub-second timing (r = .35, p = .25, Bonferroni-corrected; Fig 4a). However, as shown in Fig 4b, the magnitudes of the central tendency for sub- and supra-second intervals were not correlated for the visual modality (r = .48, p = .12, Bonferroni-corrected) or the auditory modality (r = .09, p = .99, Bonferroni-corrected; the Bonferroni corrections were applied for four comparisons). These results indicate that a modality-dependent component of the timing system is responsible for the central tendency in the sub-second range, while a common modality-independent timing system influences the central tendency in the supra-second range.

Because the reproduction task required motor responses, the reproduced intervals and corresponding analyses might be susceptible to motor noise. Wearden (2003) suggested that the motor noise lead a noisier variability of reproduced intervals for relatively shorter intervals [30]. This noise could thus make the estimates of the central tendency more unreliable for the relatively shorter sub-second intervals. One might argue that the non-significant correlation of the sub-second central tendency between modalities could be attributed to these noisy and unreliable estimates of the central tendency. To test whether the results obtained in the sub-second range were reliable, we examined whether task performance in the first and second experimental days were correlated in each condition. The magnitude of the central tendency in the first and the second experimental days were significantly correlated in all conditions (Table 1). These results suggest that the magnitude of the central tendency is stable within individuals across different experimental days, and reliably estimated in all conditions, including the sub-second intervals.

**Table 1:** Correlations between the magnitudes of the central tendency on the first and second experimental days.

Condition	Correlation coefficient	P-value
V-sub	.74	.003
A-sub	.71	.006
V-supra	.64	.010
A-supra	.66	.013

Note. V and A stand for the visual and auditory modalities, respectively. Sub and supra represent the sub-second and supra-second intervals, respectively. P-values were Bonferroni-corrected for four comparisons.

Similar to Cicchini et al.’s study, we observed a significant central tendency for the visual but not the auditory sub-second intervals. However, some participants in the present study, as well as in Cicchini et al.’s study, exhibited the central tendency for auditory sub-second intervals. One possible explanation for this finding may be the presence of individual differences in sensory precision across modalities. Temporal resolution in the auditory modality is generally finer than in the visual modality, although such auditory superiority is not always observed [31]. If the deviation of the internal representation of a stimulus interval is too small to overlap neighboring intervals, the perceived interval for that stimulus might not be affected by neighboring intervals. In both Cicchini et al.’s study and the present investigation, the spacing between stimulus intervals was the same for the auditory and visual modalities, and was not normalized by the specific timing precision across individuals or sensory modalities. Therefore, in Experiment 2, we examined whether the modality-dependent central tendency in the sub-second range resulted from differences in the temporal sensitivity between the visual and auditory systems by controlling for differences in the discrimination of sub-second intervals between the two modalities.

## Experiment 2

### Materials and Methods

#### Participants

Thirteen healthy volunteers (7 males and 6 females, 19–30 years old) participated in Experiment 2. All participants gave written informed consent for their participation in the experimental protocol, which was approved by the institutional review board at The University of Tokyo. All participants reported to have normal hearing and normal or corrected-to-normal vision.

#### Apparatus

The auditory stimuli were presented through an Audio Stream Input/Output (ASIO) compliant USB digital-to-analog converter (Roland UA-1G) and SONY MDR-XB500 headphones at 60 dB. The visual stimuli were presented on a CRT monitor (Mitsubishi Electric RDF223H, 1024 × 768 pixels, 120 Hz refresh rate). Participants were seated 57.3 cm from the monitor in a dark soundproof room; participants’ heads were stabilized using a chin rest. All apparatuses were the same as in Experiment 1.

#### Stimuli and procedure

Stimuli were the same as in Experiment 1. Visual stimuli were white disks, while auditory stimuli were pure tones (600 Hz).

Experiment 2 consisted of a discrimination task and a reproduction task. The discrimination task was first conducted to normalize individual differences in temporal discriminability across the visual and auditory modalities. In the discrimination task, three successive flashes or tones that marked two neighboring intervals were presented, and participants reported whether the first interval was longer or shorter than the second. The standard interval was always 500 ms, while the comparison intervals ranged from 350 ms to 650 ms with 50 ms steps. All comparison intervals were presented 32 times. The order of the standard and comparison intervals was randomized across trials. The visual and auditory intervals were tested in separate sessions. In the analyses, the probability at which the participant judged the comparison to be longer than the standard was plotted as a function of the comparison interval. The discrimination sensitivity was calculated by fitting a cumulative normal function as a psychometric function. The discrimination sensitivity was defined as the Weber fraction. Weber fractions were defined as the ratio of the just noticeable difference (JND; half of the difference between the intervals giving 25% and 75% of the psychometric function) to the standard interval (JND/500). Discrimination sensitivities for the visual and auditory intervals were separately estimated for each participant.

For each participant, the reproduction task followed the discrimination task in the same modality. The order of visual and auditory tasks was randomized across participants. Durations used in the reproduction task were determined for each participant and for each stimulus modality based on the participant’s performance in the discrimination task. To be precise, durations that gave 20, 35, 50, 65, and 80 percentiles in the psychometric function were used. Accordingly, all participants performed the visual and auditory tasks with physically different but perceptually the same durations.

As in Experiment 1, correct and incorrect feedback was given after each trial in the reproduction task. If the reproduced interval fell within a certain time frame of the stimulus interval, correct feedback was presented; otherwise, incorrect feedback was presented. In contrast to Experiment 1, the ratio of the width of the feedback time frame of the stimulus interval was also normalized for each participant. The feedback ratio was adaptively controlled with a one-up, one-down staircase method that added or subtracted 15% of the Weber fraction, for each incorrect or correct trial, respectively.

Trials in which the reproduced interval deviated more than 3 SDs from each condition’s mean were excluded from all analyses. As in Experiment 1, the reproduced intervals were linearly regressed to the stimulus interval, and the slopes of the linear regressions were compared across modalities as indices of the central tendency [9, 32].

### Results and Discussion

**Fig 5:**
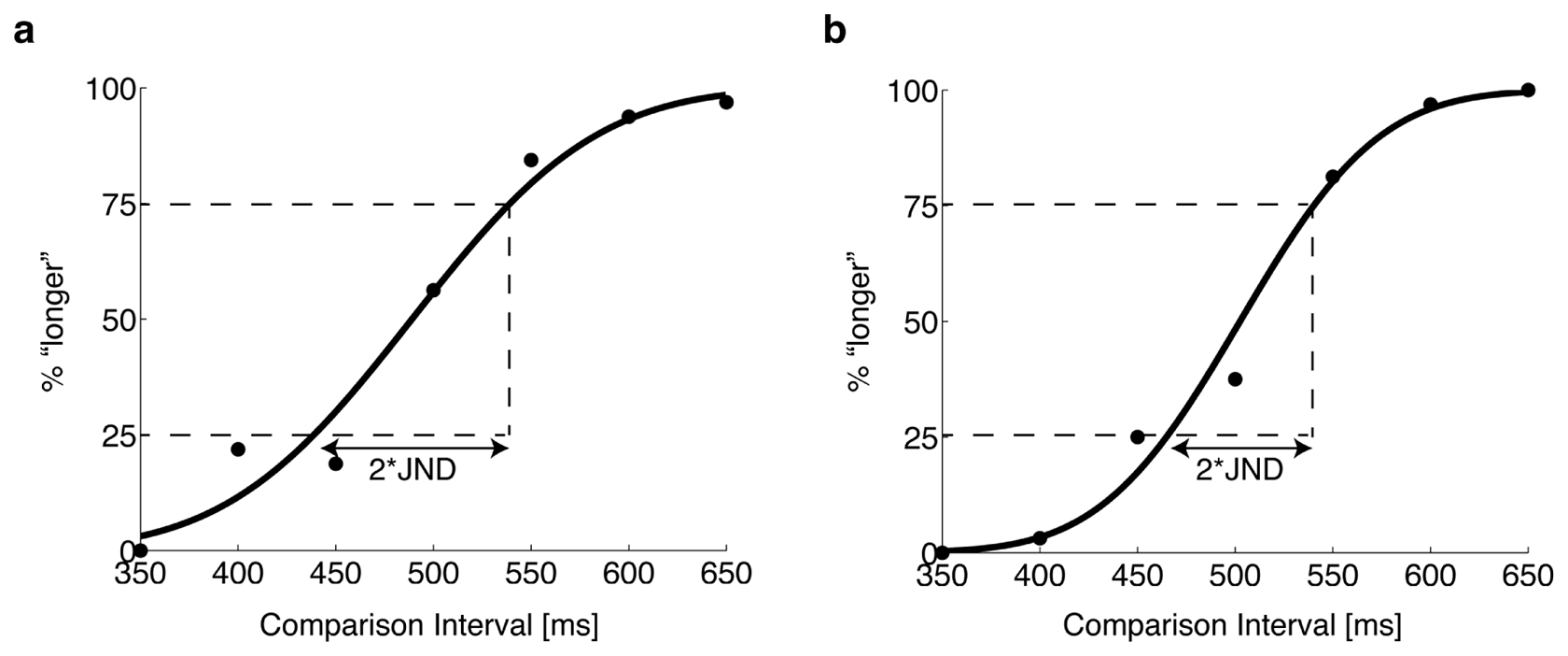
Psychometric functions of visual (a) and auditory (b) intervals for a typical subject. The probabilities at which the subject judged that the comparison (350–650 ms) was longer than the standard (500 ms) were plotted, and fitted to the cumulative normal distribution function. The discrimination sensitivity was defined as the Weber fraction, the ratio of the just noticeable difference (JND) to the standard interval.

To estimate discrimination sensitivity, we first drew psychometric functions for each participant and for each stimulus modality (Fig 5). Two participants were excluded from subsequent analyses because their response for either the shortest or the longest comparison interval did not reach 20 or 80% respectively in both modalities. The Weber fractions were 0.145 ± 0.036 for visual stimuli and 0.126 ± 0.046 for auditory stimuli. There was no significant difference between the Weber fractions of visual and auditory intervals (t(10) = 1.90, p = .09).

**Fig 6:**
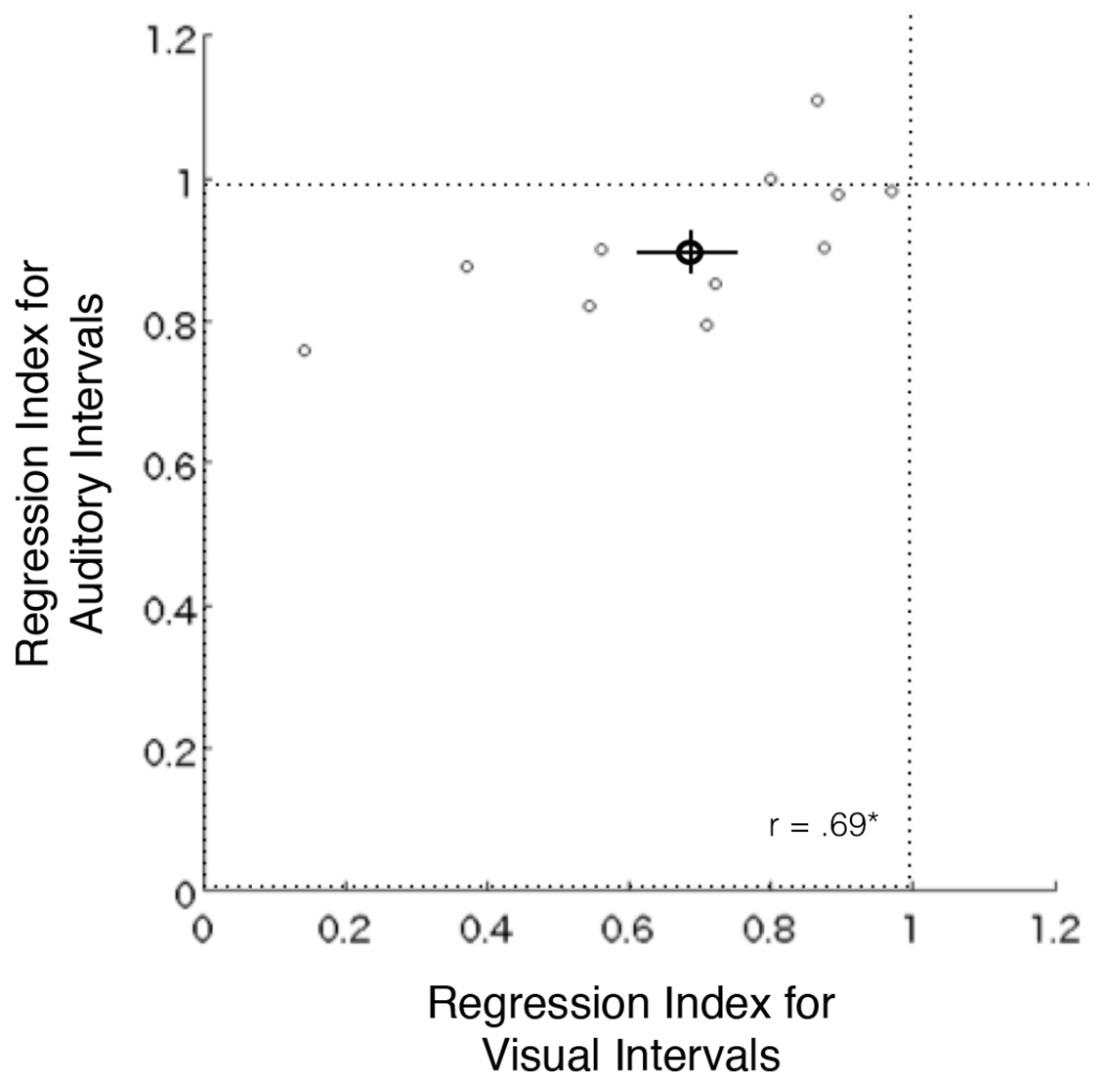
Within-individual correlations of the central tendency across visual and auditory modalities. Regression index represents the slope of the linear regression of the reproduced intervals to the stimulus intervals. Small open circles represent each individual’s data. Bold circles and bars represent the mean and the standard error of the mean, respectively. * p < .05.

In the reproduction task, even though the spacing between stimulus intervals was normalized across participants and stimulus modalities by using the discrimination task, the magnitude of the central tendency substantially varied across individuals (Fig 6). The magnitude of the central tendency was significantly larger for visual intervals than auditory intervals (t(10) = 3.89, p = .003), as in Experiment 1. Additionally, the magnitude of the central tendency for visual and auditory sub-second intervals was significantly correlated (r = .69. p = .018). This result suggests that a common or homologous system is also involved in the optimal encoding of time in both auditory and visual sub-second time perception.

In the interval discrimination task, there was no significant difference between the discrimination sensitivities of the visual and auditory modalities (Experiment 2). Kuroda et al. (2014) also reported that the sensitivity of interval discrimination is almost identical for visual and auditory stimuli [31]. Therefore, modality-dependency of the central tendency may not be explained by differences in timing precision between modalities. Because the time reproduction task requires sensorimotor timing, it is possible that the timing precision of the reproduction task and the discrimination task are independent. However, previous studies indicated that variances of the perceived intervals in the reproduction and discrimination tasks are significantly correlated [33, 34]. Therefore, it is reasonable to determine stimulus intervals in the reproduction task based on performance in the discrimination task.

In Experiment 2, a significant central tendency was also observed for auditory intervals (t(10) = 2.99, p = .014). This result is seemingly inconsistent with results in Experiment 1. One possible explanation for this difference might be the range of stimulus intervals. The means and SDs of the differences between the shortest and longest stimulus intervals was 182.1 ± 45.5 ms for visual stimuli and 160.6 ± 57.2 ms for auditory stimuli. Therefore, the stimulus intervals were narrowly ranged on average compared to Experiment 1, in which the difference between the shortest and the longest stimulus intervals was 200 ms. Previous studies have demonstrated that the distribution of stimulus intervals affects the memory bias in time perception [13, 25, 35]. Especially, the present results is consistent with the finding that the narrowly-ranged stimulus intervals amplify the central tendency in the auditory modality [26]. Further study will be needed to investigate how the distribution of stimulus intervals modulates the magnitude of the central tendency in relation to the stimulus modality and the interval discriminability.

## General Discussion

In the present study, we investigated how stimulus modality and timescale affect individual differences in the central tendency of time perception. The magnitude of the central tendency differed substantially between individuals, but was stable across different experimental days for each individual. The magnitude of the central tendency greatly varied depending on the timescale and the sensory modality, suggesting that timescale- and modality-dependent timing systems are responsible for individual differences in this phenomenon.

### Timescale-dependency of the central tendency

Our results indicate that sub- and supra-second timing influence the central tendency differently. A traditional view according the scalar property assumes that timing precision, defined as the ratio of the standard deviation of the perceived interval to the stimulus interval, is constant across sub- and supra-second ranges [15]. If the magnitude of the central tendency depends on timing precision and the scalar property, as suggested by previous studies [12, 13], then the central tendency should occur equivalently for sub- and supra-second timing. Contrary to this traditional view, the current study found that the magnitude of the central tendency was larger in supra-second timing than in sub-second timing. Several psychological studies support this finding and have also demonstrated that timing performance is less precise in the supra-second range compared to the sub-second range [20, 36]. The larger central tendency in the supra-second range might result from the noisier representation of intervals in the supra-second range. In addition to the difference in timing precision between sub- and supra-second timing, it should be noted that the experimental setting of ITIs might also lead the larger central tendency in the supra-second range. Meck (1985) suggested that the timing performance is impaired when the ratio of the timed interval to the ITI is large [37]. In the present study, the same ITIs were used for sub- and supra-second trials. Therefore, the ratio of the timed interval to the ITI was larger in the supra-second, which might lead the larger timing bias in the supra-second range.

The magnitudes of the central tendency in the sub- and supra-second range were not correlated in both modalities (Experiment 1). Therefore, no evidence for the involvement of timescale-independent timing mechanisms in the central tendency was found. This result is consistent with a previous investigation that showed timing precision was not correlated between sub- and supra-second intervals [19]. In the present study, stimulus intervals within a given session had narrow ranges (i.e., 0.4–0.6 s for sub-second, 2.0–3.0 s for supra-second intervals) in order to investigate the difference in the central tendency between sub- and supra-second timing. In contrast, previous studies, including the original work of Vierordt (1868), used wide intervals that spanned the sub- and supra-second ranges within an experimental session, and observed overestimation of short sub-second intervals and underestimation of long supra-second intervals [7]. If the sub- and supra-second timing systems were completely independent, the central tendency would not occur across different timescales, but this was not the case. Rammsayer & Troche (2014) proposed a hierarchical mechanism of time perception in which a timescale-independent superordinate processing system controls the sub- and supra-second timing mechanisms [18]. Based on the present study, timing precision in the sub- and supra-second ranges affects individual differences in the magnitude of the central tendency. These noisy interval representations in modality-dependent timing systems may be further processed by the timescale-independent superordinate processing system.

### Modality-dependency of the central tendency

In the present study, the central tendency was modality-dependent in the sub-second range, but not in the supra-second range; both modalities exhibited a comparable central tendency in the supra-second range. These results suggest that a common modality-independent timing system regulates the central tendency in the supra-second range, while a modality-dependent timing system has a greater impact on the central tendency in the sub-second range.

A previous study investigated the central tendency in the supra-second range in the visual and auditory modalities, and came to the opposite conclusion that a modality-dependent timing system does impact the central tendency in the supra-second range (Noulhiane et al., 2009). In the central tendency, short intervals are overestimated and long intervals underestimated, resulting in an “indifference point” where durations are estimated accurately. Noulhiane et al. (2009) found that this “indifference point” was different between the visual and auditory modalities when identical stimulus intervals were tested [26]. In contrast, Ryan (2011) showed that the distortion pattern of reproduced intervals was comparable between the visual and auditory modalities [25]. In the present study, we observed high within-individual correlations between the magnitudes of the central tendency in the visual and auditory modalities in the supra-second range. This result strongly suggests the involvement of a common modality-independent timing system that regulates the central tendency in the supra-second range. However, this result does not necessarily exclude the possibility of a modality-dependent mechanism in the regulation of supra-second time perception. Consistent with the findings of Noulhiane et al. (2009), we observed that auditory intervals were overestimated more than visual intervals in the supra-second range. This overestimation of auditory intervals suggests there are different “indifferent points” between the visual and auditory modalities, and makes it appear as if the magnitude of the central tendency is different for visual and auditory stimuli. However, this general overestimation of all auditory intervals could be independent of the central tendency, which is generally thought to be the result of the overestimation of short intervals and the underestimation of long intervals. Further investigation is necessary to ascertain how modality-dependent general overestimation occurs in the supra-second timing system.

In the sub-second range, differences in the central tendency between the visual and auditory modalities remained, even when the ability to discriminate intervals was controlled across sensory modalities (Experiment 2). Previous studies have shown that auditory modality dominates the time perception for audio-visual stimuli [38, 39]. These studies suggest that the timing system utilizes auditory information for temporal estimation rather than visual, even when the perceptual threshold is equalized across the visual and auditory modalities. In contrast to these studies, the present study suggests that sub-second timing in the auditory modality largely relies on stimulus input, and is less affected by contextual information, such as previously presented intervals. However, this does not mean that the auditory sub-second timing is not affected by contextual information. Various studies have indicated that previously presented intervals do affect perceived auditory intervals [10, 40, 41]; furthermore, the present study also observed a significant central tendency for auditory intervals in Experiment 2.

Interestingly, we observed a significant correlation in the central tendency between the visual and auditory sub-second intervals in Experiment 2, in which the spacing between stimulus intervals was normalized across modalities based on timing precision that was measured by the interval discrimination task. Therefore, the modality-independent timing mechanism might also be involved in the central tendency in sub-second timing, in addition to the modality-dependent timing mechanism. Several studies support this conclusion that modality-dependent and modality-independent components influence the sub-second timing system. For example, a psychophysical and modeling study revealed a hierarchical timing mechanism whereby modality-specific processing occurs first, followed by modality-independent processing [17]. An alternative model assumes that temporal information is primarily encoded in the auditory system [42, 43]. In this model, temporal information from all sensory modalities is transformed into an auditory format for temporal processing. These two models are not mutually exclusive, and both are consistent with the present results. Further study is necessary to examine the interaction between sensory modalities.

In summary, the present study suggests that individual differences in the central tendency might be associated with a common modality-independent timing mechanism for supra-second timing, and with both modality-dependent and modality-independent timing mechanisms for sub-second timing.

### Incorporating timescale- and modality-dependent components into computational models of the central tendency

In the present study, we showed that both timescale and modality influenced the central tendency. These findings have important implications for the creation of computational models of the central tendency. Recent computational models have accounted for the central tendency in the context of a Bayesian framework [12, 13]. Bayesian models assume that a noisy representation of the current stimulus interval (likelihood) is combined with a prior representation of the probability distribution of the stimulus intervals presented within the experimental session. Both the likelihood and the prior determine a posterior distribution of the perceived interval, and participants make responses based on this posterior distribution. Such models have two fundamental assumptions regarding the probability distribution of the likelihood that need to be altered in light of the current findings.

One assumption is that the mean of the likelihood is equal to the stimulus interval. However, previous studies have shown that auditory intervals tend to be estimated longer than visual intervals [44, 45], which demonstrates that the perceived interval can be systematically shifted from the physical stimulus interval. In the present study, we also observed overestimation of auditory intervals in the supra-second range. Previous Bayesian models cannot account for these constant errors. One possible explanation for the overestimation of auditory intervals is that auditory signals drive the internal clock faster than visual signals [10, 44]. However, the timed intervals in the present study were empty intervals that were defined by two brief sensory events and have no signal during the interval itself for both visual and auditory conditions. Considering the absence of sensory signal during the timed interval, it might be unlikely that the observed overestimation for auditory intervals is ascribed to the faster accumulation of the internal clock throughout the timed interval. Another interpretation for the overestimation of auditory intervals is that the latency to direct attention to the interval onset might be different between modalities, that is, the auditory signal might grab the attention faster [44, 46]. Since the likelihood of timed intervals could change in either case [5], the modality-dependent constant errors should be explained in the framework of Bayesian models by allowing the mean of the likelihood to shift from the physical stimulus interval.

The other assumption regarding the distribution of the likelihood that should be addressed is the scalar property. Recent psychophysical studies have reported that the scalar property is violated under certain conditions, and that timing precision changes around one second [20, 47]. Indeed, we revealed that supra-second timing is more susceptible to the central tendency than sub-second timing, suggesting that timing precision is lower in the supra-second range than in the sub-second range. Therefore, caution should be taken when assuming that the scalar property holds for all intervals across different timescales. The noise distribution of the likelihood may need to be determined separately for sub- and supra-second intervals. Therefore, future studies should explore whether the interval distributions within the same timescale and across different timescales exhibit quantitatively similar central tendencies or not. These approaches will provide the means for identifying sources of timing noise that mediate the central tendency and establish a comprehensive model for the optimal encoding of time.

The present study demonstrated that the internal representation of time is subject to a context-dependent sensori-motor process that optimally encodes temporal information in a modality- or timescale-dependent manner. Although the present study focused to time reproduction as in previous studies [9, 12, 13, 48], Petzschner and her colleagues indicated that we have a common Bayesian principle for optimal magnitude estimation such as movement distance, stimulus length, and also interval time [49, 50]. The modality- and timescale-dependence of the central tendency observed in the present study suggests that the source of timing noise can be unique to each timescale or each sensory modality, and then, a common computational process realizes the statistical optimality context-dependently for such noisy representation of time.
